# Adeno-Associated Virus Expression of α-Synuclein as a Tool to Model Parkinson’s Disease: Current Understanding and Knowledge Gaps

**DOI:** 10.14336/AD.2021.0517

**Published:** 2021-07-01

**Authors:** Taylor E Huntington, Rahul Srinivasan

**Affiliations:** ^1^Department of Neuroscience & Experimental Therapeutics, Texas A&M University College of Medicine, 8447 Riverside Pkwy, Bryan, TX 77807, USA.; ^2^Texas A&M Institute for Neuroscience (TAMIN), College Station, TX 77843, USA

**Keywords:** Parkinson’s disease, alpha-synuclein, dopaminergic, model

## Abstract

Parkinson’s disease (PD) is the second most common neurodegenerative disorder in the aging population and is characterized by a constellation of motor and non-motor symptoms. The abnormal aggregation and spread of alpha-synuclein (α-syn) is thought to underlie the loss of dopaminergic (DA) neurons in the substantia nigra pars compacta (SNc), leading to the development of PD. It is in this context that the use of adeno-associated viruses (AAVs) to express a-syn in the rodent midbrain has become a popular tool to model SNc DA neuron loss during PD. In this review, we summarize results from two decades of experiments using AAV-mediated a-syn expression in rodents to model PD. Specifically, we outline aspects of AAV vectors that are particularly relevant to modeling a-syn dysfunction in rodent models of PD such as changes in striatal neurochemistry, a-syn biochemistry, and PD-related behaviors resulting from AAV-mediated a-syn expression in the midbrain. Finally, we discuss the emerging role of astrocytes in propagating a-syn pathology, and point to future directions for employing AAVs as a tool to better understand how astrocytes contribute to a-syn pathology during the development of PD. We envision that lessons learned from two decades of utilizing AAVs to express a-syn in the rodent brain will enable us to develop an optimized set of parameters for gaining a better understanding of how a-syn leads to the development of PD.

Parkinson’s disease (PD) is the second most common neurodegenerative disorder, affecting 1-2% of the population over 65 years. Clinically, PD is characterized by a loss of dopaminergic (DA) neurons in the substantia nigra pars compacta (SNc) and the onset of motor symptoms such as bradykinesia, resting tremors, postural instability, and muscle rigidity. Apart from motor symptoms, PD is also inundated with non-motor symptoms that occur before and during motor dysfunction, such as sleep disturbances, constipation, heightened sensitivity to pain, anxiety, depression, and cognitive dysfunction [1, 2]. These multifaceted clinical features correlate with a sequential degeneration of neurons within several discrete loci of the brain, which points to both spreading neuropathology and regional vulnerability as underlying causes for PD [3-7].

Rather than a single source of neurodegeneration, PD likely results from a few select factors with central roles in triggering the cascade of clinical presentations and spreading neuropathology. In this regard, the accumulation of Lewy bodies and Lewy neurites in the nigrostriatal pathway is a classic hallmark of clinical PD[8]. These distinctive histopathological features consist of an aggregation of ~40 different proteins with alpha-synuclein (a-syn) at its core[8]. Importantly, autopsies from PD patients with surgical grafts of mesencephalic DA neurons into the striatum show Lewy body inclusions that spread from the host tissue into exogenous DA grafts [9]. Together, these findings strongly implicate abnormal a-syn aggregation as an important nucleating factor for neurodegeneration during PD.

The idea that a-syn is a central player in the pathogenesis of PD has led to the development of molecular tools focused on understanding the mechanisms underlying a-syn-mediated neuro-degeneration with the goal of discovering potential a-syn targeted therapeutics for PD. Consequently, at least three different tools have been developed to model the pathological features of a-syn in rodents. These tools include transgenic mice overexpressing a-syn, pre-formed a-syn fibrils (PFFs), and viral mediated overexpression with either adeno associated virus (AAV)- or lentivirus (LV) expressing a-syn. Each of these tools model distinct aspects of a-syn pathology *in vivo*, and recapitulates select symptoms of human PD.

In this review, we specifically focus on the use of AAV-mediated expression of a-syn as a tool to model PD *in vivo*. Over the past two decades, AAVs expressing a-syn have provided an effective means to model the progressive loss of DA neurons in rodents and appear to model both early and late features of PD pathogenesis. However, studies from various groups using AAVs expressing a-syn have shown a range of outcomes from very slight to greater than 90% DA loss, leading to numerous choices when selecting the most appropriate parameters to model PD pathogenesis in rodents. We provide a detailed account of the observed differences in published studies, along with an informed perspective on how one could approach the use of AAVs to model PD in rodents. We also provide an overview of specific mechanisms by which a-syn can induce astrocytic dysfunction and DA loss during PD, which makes AAVs a valuable tool for expressing a-syn in astrocytes of the rodent brain.

## 1. AAV-mediated a-syn overexpression in rodents as a tool to model PD

The first step towards implicating a-syn in PD pathogenesis was the identification of a case of familial PD due to triplication of the *SNCA* gene that codes for a-syn [10]. This finding was soon followed by the discovery that a-syn is a main component in Lewy bodies and Lewy neurites, which led to focused studies on the role of a-syn in PD [11].

**Table 1:** Table of studies utilizing unilateral AAV induction into the SNc of rodents for recapitulation of Parkinson’s disease.

Ref.	Animal/Sex	Inj. Age (wks)	Serotype	Promoter	A-syn Strain	Enhancers	Viral Load (gc)	Length of expression (wks)	TH loss (SNc)	TH loss (STR)	DA loss (STR)
[16]	SD Rats - F	*8-12	AAV2/1	CAG	A53T	WPRE & bGF-polyA	3.40E+09	3 6	None -28%	-7% -24.5%	None -43%

[22]	C57BL/6 - M	*8-10	AAV2/1	CMV	WT	--	6.20E+08	4 8 12	-- -34% -50%	-10% -25% -45%	-10% -20-30% -50-60%

[70]	C57BL/6 Pups	P0	AAV2/1	CAG	WT	WPRE	2.70E+10	4 12 24	--	--	--

[24]	C57BL/6 - M	12	AAV2/1	CAG	A53T	WPRE & bGF-polyA	7.74E+09	10	-30%	-20%	-38%

[13]	SD Rats -M	12	AAV2/2	CAG	A30P	--	3.00E+10	52	-53%	--	--

[14]	SD Rats -M	10	AAV2/2	CMV	WT	--	1.26E+09	13	-49%	--	-10%

[15]	C57BL/6 - M	12-16	AAV2/2	CAG	WT	WPRE	2.67E+08	4 12 24	-- -5-15% -25%	none none none	--

[88]	C57BL/6 - M	8-12	AAV2/2	CAG	WT	WPRE	1.30E+10	2 4 12	--	--	--

[79]	Rats	--	AAV2/5	CAG	WT	--	1.40E+10	4 8 26	-- -40% -60%	--	none -36% -64%
					S129A		1.40E+10	4 8 26	-70% -70% -60%	--	none -50% -70%
					S129D		1.40E+10	4 8 26	none none none	--	none none none

[125]	SD Rats - F	adult	AAV2/5	CAG	WT	--	6.07E+10	3 8 15	none none none	none none none	none -10-15% -20%
					α-synΔ110	--	8.25E+10	3 8 15	none none none	none none -35-40%	none -10-15% -40%
					WT & α-synΔ110	--	6.07E+10 & 8.25E+10	3 8 15	none -15% -36%	none -25-30% -35-40%	none -30% -35%

[18]	SD Rats - M	--	AAV2/5	CAG	WT	--	2.00E+10	4 8 12	-35% -60% --	-- -42% --	--
							2.00E+11	4 8 12	-70% -90% --	-42% -- --	--

[87]	SD Rats - F	*8-12	AAV2/5	CAG	WT	--	2.00E+10	4 8 15	none -56% -54%	none -50% -60%	--
					S129A		4.00E+10	4 8 15	none -44% -44%	none none -40%	--
					S129D		4.00E+10	4 8 15	-40% -49% -49%	none -40% -40%	--

[81]	Wistar Rats - F	*8-12	AAV2/6	CMV	WT	--	3.40E+08	8	-20%	†--	--
					WT S129A		2.40E+08	8	-70%	--	--
					WT S129D		3.40E+08	8	-10%	--	--
					A30P		1.80E+08	8	-20%	--	--
					A30P S129A		3.80E+08	8	-70%	--	--
					A30P S129D		2.80E+08	8	none	--	--

[17]	SD Rats - F	*8-12	AAV2/6	h-syn	WT	WPRE	1.11E+10	8	-66%	-68%	--

[64]	SD Rats - F	*8-12	AAV2/6	h-syn	WT	WPRE	3.10E+08	10 days 3 wks 5 8 16	none -42% -60% -80% -75%	-20% -30% -30% -59% -60%	none -20% -31% -56% -61%
				CAG		--	3.10E+08	10 days 3 wks 5 8 16	-- none -- none --	-- none -- none --	--

[69]	SD Rats - F	*8-12	AAV2/6	h-syn	WT	WPRE	2.30E+11	3 8	none -43%	-- -31%	--

[25]	SD Rats - F	*8-12	AAV2/6	PGK	WT	--	1.50E+07	14	-45-50%	-25-30%	-50%

[62]	SD Rats - F	16	AAV2/6	h-syn	WT	WPRE	1.11E+10	8	-50%	--	-60%

[71]	SD Rats - F	*8-12	AAV2/6	h-syn	WT	--	8.80E+08	7 16 28	-10% -30% -50%	-20-25% -30% -40-50%	--

[65]	Wistar Rats - F	*8-12	AAV2/7	CMV-h-syn	--	A53T	9.00E+08	15 days 29 days 11 mo	-- -80% --	--	-60% -92% --

[19]	C57BL/6 - F	8	AAV2/7	CMV-h-syn	WT	WPRE	5.20E+08	5 days 4 wks 8	-- -19% -23%	-- -17% -56%	--
					WT		8.00E+08	5 days 4 wks 8	-- -45% -50%	-- -56% -86%	--
					WT		1.60E+09	5 days 4 wks 8	-- -57% -82%	-- -73% -86%	--
					A53T		8.00E+08	5 days 4 wks 8	-- -51% -59%	-- -57% -91%	--

[23]	SD Rats - M	8-10	AAV2/9	h-syn	WPRE	WT	4.00E+09	1 4	-- -57%	-- -60%	-- -70%

[66]	C57BL/6 - M	8-10	AAV2/9	CMV-h-syn	A53T	--	8.40E+08	20	-30%	-25-30%	--
	SAMR1 - M								none	-30%	--
	SAMP8 - M								none	-30%	--

[190]	SD Rats - F	8	AAV2/9	CAG	A53T	--	1.16E+10	10	-55%	--	--

[191]	Fisher 344 Rats - M	12	AAV2/9	CAG	WT	WPRE & bGF-polyA	4.40E+09	2 4 16	none -25-30% -25-30%	--	--

When a study did not report a parameter (--) is used. When a study reported either no or non-significant changes in a parameter, none, is used. Columns/Abbreviations: Ref. (reference), rodent strain and sex (M/F), age at injection, AAV serotype, promotor, strain of a-syn, enhancing elements, viral load injected in total genomic copies, length of expression of a-syn, loss of tyrosine hydroxylase (TH) neurons in the SNc, TH loss in the striatum, dopamine (DA) loss in the striatum.

* age as determined by average weight reported.

† As measured by TH and VMAT2.

### Biochemical and molecular changes

Based on amino acid (aa) composition, a-syn has three distinct domains, with an amphipathic N-terminal domain (aa 1-60), a central hydrophobic domain (aa 61-94), and an acidic C-terminal domain (aa 95-140). a-syn mainly localizes to pre-synaptic terminals [20, 21], where the amphipathic and central domains mediate a-syn interactions with cellular membranes. These interactions result in distinct biochemical and molecular changes during PD.

AAV-mediated a-syn expression in rodents causes a paradoxical increase in dopamine turnover along with a depletion of the dopamine transporter, DAT [16, 17, 22-25]. In addition, a dynamic change in DAT binding occurs between 3 and 6 weeks post injection, beginning with a 31% increase and ending with a 48% decrease in DAT binding [16]. This would suggest that early stages of a-syn pathology may be associated with a paradoxical increase in striatal dopamine. Indeed, 4-month old male transgenic mice overexpressing a-syn under the Thy-1 promoter also show a surge in dopamine release within the striatum[26]. Following this initial surge in striatal dopamine, rodents show a loss of the axonal terminals of SNc DA neurons in the striatum [26], which is accompanied with a decrease in striatal dopamine as well as its major metabolites, 3,4-dihydroxyphenylacetic acid (DOPAC) and homovanillic acid (HVA). These findings are similar to previous studies in patients with PD showing an initial degeneration of DA axons in the striatum [27], and relates to an increased risk of PD in humans with DAT mutations [28]. This suggests that AAV-mediated a-syn expression in the rodent SNc reliably models clinical aspects of PD related to fluctuations in striatal dopamine.

Dysfunctions in striatal dopamine also trigger increases in burst firing within the subthalamic nucleus (STN). In PD patients, this is typically seen during surgical implantation of deep brain stimulation electrodes into the STN and is also noted in PD toxin models [29-32]. One study overexpressing a-syn using AAV2/5-CAG-WT-a-syn-WPRE in the SNc has recapitulated heightened burst firing within the STN of female Sprague Dawley (SD) rats [33], suggesting that AAV-mediated a-syn pathology in the SNc can have wide-ranging consequences that lead to dysfunction of other basal ganglia nuclei.

Within the pre-synaptic compartment, a-syn interactions with SNARE protein complexes as well as with the vesicular transporter of monoamines (VMAT2) have been described [34-37], suggesting a primary role for a-syn in the trafficking and assembly of synaptic vesicle complexes[38-40]. In support of this idea, all known point mutations in a-syn associated with familial PD (A30P, E46K, H50Q, G51D, A53T, and A53E) localize to the N-terminus, suggesting that alterations in the ability of a-syn to bind to cellular membranes may underlie its toxic effects, and trigger abnormal aggregation of a-syn [41-50].

Interactions between a-syn and multiple cellular and subcellular targets[34, 51, 52] are also mediated by the core region of this intrinsically disordered protein. The central, hydrophobic region of a-syn (aa 61-94) was first identified in amyloid plaques of Alzheimer’s disease patients [44]. Termed the non-amyloid-β component (NAC), conformational changes in this region are responsible for a-syn fibril formation and aggregation due to crosslinking of β-sheets [53-57]. Indeed, the idea that a-syn may exist in a dynamic equilibrium between a disordered monomer and a tetramer under physiological conditions [58-61] would indicate that changes in both regions could abnormally alter monomer:tetramer a-syn ratios. This may be due to either an inability of a-syn to bind to membranes, an abnormal increase in a-syn binding to membranes or result in abnormal a-syn aggregates and the formation of Lewy pathology. Further understanding of a-syn structure in humans and in AAV-mediated expression in rodents are needed to determine the full relationship of a-syn structure and pathology in PD.

### Behavioral changes

The early pre-symptomatic stage following AAV-mediated expression of a-syn in rodents correlates with early striatal dysfunction [17, 22, 62, 63], which could precede non-motor behavioral changes reminiscent of prodromal symptoms such as anxiety or depression. During the symptomatic stage, the extent of DA neuron loss and consequent deficits in striatal dopamine are large enough to induce motor dysfunction [12, 13, 18, 19, 23, 64-67], while the more advanced stages are likely characterized by an increase in mortality. Therefore, depending on the exact model, AAV-mediated a-syn expression in the SNc can recapitulate important aspects of early, mid as well as late stages of clinical PD.

The most widely tested behavior among all PD models is locomotion. In AAV models with a unilateral injection of AAV-a-syn, behavioral paradigms that focus on measuring motor asymmetry such as amphetamine or apomorphine induced rotations, cylinder test, or paw-stepping test are commonly employed [13]. In addition, the beam walk, open field, and rotarod test are also common behavioral tests for AAV-a-syn models [18, 19, 23, 65, 66, 68-70]. Although the vast majority of AAV studies show motor deficits compared to controls, there is variability in the amount of SNc dopaminergic loss when correlated with motor symptoms. A majority of AAV-based studies report motor deficits only when greater than 50% of SNc DA neurons are lost, which is consistent with clinical PD [12, 13, 17-19, 23, 65-67]. However, several other studies show motor deficits with less than 40% SNc DA neuron loss [16, 17, 24, 25, 68, 71, 72]. As shown in Table 1, potential sources for this variability are numerous and could include differences in behavioral assays, in the methodology used for quantification of PD-related behaviors, the AAV serotype, length of time for expression of a-syn or species of rodent. Studies focused on systematically comparing one or more of these parameters are therefore essential for developing a more consistent and standardized AAV-based a-syn expression model for PD in rodents.

An array of non-motor symptoms appears prior to motor dysfunction and are attributed to both DA and non-DA neuron pathology. These symptoms include sleep disturbances, depression, anxiety, cognitive dysfunction, heightened pain, hallucinations, impaired color vision, and hyposmia [1, 73-75]. A few AAV a-syn models in rodents have assessed non-motor symptoms in PD [18, 67-69]. Male AAV-a-syn SD rats show significant loss in odor discrimination at 9 weeks, followed by significant deficits in rotarod performance [67]. In a recent study, female SD rats unilaterally injected with AAV-a-syn showed anxiety, but no cognitive deficits. A similar anxiety phonotype has been reported in male Wistar rats with bilateral SNc injection of AAV a-syn [68]. Additionally, gastrointestinal (GI) disturbances have been associated with a-syn pathology in the gut-brain axis [76, 77], but only two studies so far have explored the ability of AAV-a-syn in modeling this feature of PD [78]. Thus, AAV-mediated expression of a-syn in the rodent SNc appears to model important aspects of PD such as anxiety, and GI dysfunction. However, not all prodromal symptoms may be present in AAV a-syn PD models. Further characterization of non-motor symptoms in PD such as depression, cognitive dysfunction and hyposmia is necessary. This would be especially beneficial when investigated with AAV-a-syn models already shown to reproduce DA neurodegeneration and motor symptoms.

### Phosphorylated a-syn (p-a-syn)

The C-terminus of a-syn (aa 96-140) is a highly unstructured region with several post-translational modification sites, including phosphorylation sites (Tyr-125,133,136 and Ser-129). This region is populated with negatively charged amino acids resulting in a random coil structure at the tail of a-syn. The most common post-translational modification is phosphorylation of a-syn at S129 (p-a-syn). Interestingly, p-a-syn is expressed throughout the brain in PD patients, and in PFF and AAV a-syn models of PD [67, 79, 80]. This suggests that phosphorylation of S129 is pathological. In accordance with this idea, abundant expression of p-a-syn is accompanied by bulging neurite morphology in the striatum and a dystrophic morphology of cell bodies in the SNc of AAV a-syn expressing rodents [19, 33, 65, 70]. Further evidence for a pathological role of p-a-syn comes from the finding that p-a-syn comprises greater than 90% of LBs seen in post-mortem human tissue. Attempts in AAV studies to further characterize p-a-syn inclusions consist of proteinase K digestion, immuno-staining for ubiquitin or thioflavin S, or a combination [16, 24, 67, 71, 72, 80-83]. A recent study bilaterally overexpressing A53T a-syn demonstrated a shorter half-life for p-a-syn and increased concentrations of p-a-syn with proteasome inhibition, indicating p-a-syn as a favored substrate in the ubiquitin-proteome system [84]. Additionally, lysosome and autophagosome-like structures demarcated in the inner architecture of LBs suggest dysfunction in degradation pathways [85].

Rodent models of AAV-a-syn expression provide the advantage of expressing a-syn with aa substitutions that mimic either a phosphorylated (S129D) or a dephosphorylated (S129A) form of the protein. The dephosphorylated state of S129, in some AAV rat models, correlates with higher rates of DA cell death in both WT and A30P strains [79, 86]. However, another study using a different rat strain reported slower DA cell loss with S129A expression [87]. In order to better understand the role of S129 a-syn phosphorylation in PD, differences in experimental parameters such as AAV serotype, viral titers, and strain of rodents used will need to be systematically addressed in a standardized setting.

### Neuroinflammation

In AAV a-syn models, the overexpression of WT or mutant a-syn activates microglia and induces lymphocyte infiltration. Neuroinflammatory markers such as IL-6, TNF-α, and CD68 are upregulated, along with production of CD8^+^ and CD4^+^ T lymphocytes [65, 72, 80, 88, 89]. Interestingly, partial rescue of DA neurodegeneration occurs with pharmacological inhibition of upregulated angiotensin II type I receptors in an AAV α-syn rat model [23]. While microglia are the resident immune cell in the CNS, recent evidence points to a larger role for astrocytes in neuroinflammation than previously thought, particularly with regard to a-synucleinopathies [90, 91]. It is important to note that although AAVs provide a useful model for studying neuroinflammation during PD, GFP-expressing AAVs injected into the rodent hippocampus have been shown to induce astrocyte reactivity at high titers [92]. Therefore, one has to be cautious with regard to the serotype and titer of the AAV being used to express a-syn. In summary, rodent models utilizing AAV-mediated a-syn expression parallel several aspects of PD pathology including neurochemical changes, non-motor and motor behaviors, and neuroinflammatory changes in the brain.

### Changes in organelle function

Apart from the pre-synaptic terminal, potent membrane binding capabilities of a-syn have also been identified in organelles such as mitochondria [38, 52, 93, 94], the endoplasmic reticulum [95, 96], the Golgi apparatus[97-99] and the nucleus [100]. Multiple forms of a-syn disrupt protein synthesis and degradation pathways, restrict intracellular calcium handling, increase oxidative stress, and impair phagocytic activity[52, 101].

AAV a-syn models demonstrate aggregates localizing to mitochondria associated membranes [38, 39]. This disrupts Ca^2+^ handling from ER to mitochondria and ATP production, and alters energetic homeostasis in the brain [102, 103]. Additionally, a-syn interactions with calcium-ATPases lead to fragmentation in the Golgi apparatus [52]. Mutations in genes implicated in PD have direct consequences on mitochondrial function, but are exacerbated with accumulation of a-syn aggregates [104, 105]. In neurons, region specific decreases in respiratory chain complexes correlate with idiopathic PD in humans [103]. However, another study showed higher levels of respiratory complexes without a change in the number of neuronal mitochondria [106]. a-syn further elicits subcellular damage in the nucleus via interactions with DNA and histones that are thought to impact production of vesicular transport machinery [52]. Furthermore, a-syn degradation is mediated by the ubiquitin-proteasome system and the autophagy-lysosomal system; however, recent studies have revealed damages to these systems that may encourage a-syn aggregation [83, 84, 107, 108]. Taken together, these reports suggest diverse roles for a-syn in subcellular function with the potential for abnormal a-syn to exert global pathological effects on neuronal physiology. The vast majority of organelle interactions have been studied in neurons [5, 52], but emerging evidence shows parallel mechanisms of a-syn induced pathology in glial organelles, including astrocytes [109, 110]. Understanding how these mechanisms affect PD pathogenesis will greatly enhance our knowledge of PD, and neurodegeneration in general.

## 2. An analysis of AAVs as viral vectors for expressing a-syn

Recombinant AAVs are multi-functional tools in research and are widely used for gene delivery to the central nervous system (CNS) of rodents as well as humans [27, 111]. Advantages of recombinant AAVs include their low immunopathogenicity and the episomal expression of transgenes, with a very low probability of integration into the host genome [112]. Moreover, the ability of AAVs to transduce slow-dividing or non-dividing cells in a targeted manner is beneficial for expressing proteins in different cell types within the CNS. Together, these features make AAVs an attractive tool to express a-syn as a means to understand how pathological a-syn initiates PD. As shown in Table 1, key elements of AAVs that vary among rodent a-syn studies include the genome with promoter and enhancer elements, capsid serotype, the strain of a-syn encoded, method of purification, and determination of functional titer. In addition, the choice of control AAVs for experiments along with the species, strain, age, and sex of rodents significantly influence the outcome of studies in terms of the extent of SNc DA neurodegeneration. In the next sections, we consider each of these elements in some detail.

### AAV genome

Recombinant AAVs are classified based on their genomic sequence and capsid serotype. Thus, AAV2/5 denotes a recombinant AAV with an AAV2 genomic sequence and an AAV5 capsid serotype. The AAV genome typically consists of a transgene cDNA cassette and a polyadenylation signal driven by a promoter for tissue/cell specific expression. Enhancers and/or post-transcriptional elements can be added within the cassette for optimized transgene expression. AAV expression systems are compatible with a wide range of minimal promoters and enhancers for transgene expression. This not only increases flexibility in altering a-syn expression, but also gives rise to increased variability in modeling a-syn pathology. Some common minimal promoters used in AAV a-syn models include the cytomegalovirus (CMV), chicken beta-actin (CBA), human synapsin I (hSyn or Syn-1), phosphoglycerate kinase 1 (PGK), cytomegalovirus-enhanced synapsin I (CMVie/hSyn), and the chicken beta actin/cytomegalovirus enhancer hybrid (CAG). Although these promoters efficiently transduce DA neurons in the SNc[17, 65, 113], how these promoters influence the subsequent cell-to-cell transfer of a-syn has not been completely elucidated. In addition to minimal promoters, the Woodchuck Hepatitis Virus Posttranscriptional Regulatory Element (WPRE) is commonly included in AAV-a-syn expression systems. WPRE consistently produces robust dopaminergic loss compared to AAVs with no WPRE [64]. On the other hand, the presence of WPRE does not ensure cell death as other studies report lower dopaminergic degeneration despite higher injected viral titer [24, 69]. Thus, although promoter and enhancer elements can influence the outcome of experiments with a-syn expression, the mechanisms underlying these variations remain to be determined.

### AAV capsid

In rodents, AAV2/2 shows low transduction efficiency; however, studies employing AAV 2/1, 2/5, 2/6, 2/7, 2/8, and 2/9 in the CNS show a marked improvement in terms of transduction efficiency [114-118]. In a separate study, Aschauer and colleagues determined that AAV 2/5, 2/8, and 2/9 show increased transgene expression in striatum, hippocampus, and auditory cortex of adult mice when compared to AAV 2/1 [119]. Furthermore, transduction efficiencies of AAV 2/1 and 2/5 are higher in adult mouse tissue than in neonatal brains, and transduction efficiency has been shown to change over time in neonatal mice [116, 118, 120]. Apart from the AAV serotype, the species of rodent used (mice versus rats) may play a role in determining AAV expression efficiency. Interestingly, models utilizing AAV 2/1 and AAV2/7 show dose dependent nigral cell death with WT and A53T a-syn overexpression in rodents [16, 19]. However, a comparison shows AAV 2/1 expressing the A53T mutant a-syn promotes greater DA degeneration in rats than in mice[16, 24]. These unknown interactions between AAV serotypes and rodent species point to species-specific differences that may include either permissivity to AAV infection, immune responses, or genetic differences between mice and rats.

Apart from influencing the rate of DA degeneration *in vivo*, AAV capsid serotypes can alter other factors such as transduction volume, cell tropism, and even the retrograde transport of AAV-expressed proteins [121, 122]. While AAVs are able to transduce larger areas of tissue than PFFs, a lower transduction volume with AAV2/8 was reported in rats [114]. This appears to be a function of the AAV2/8 serotype since a lower AAV2/8 transduction volume was also observed in adult mouse brains; however, this deficit disappeared with an increase in viral titer [116]. Interestingly, some AAVs may be more efficient at the retrograde transport of AAV-expressed proteins in neurons. In a study comparing seven different serotypes, only AAV2/5 was able to transduce SNc neurons after striatal injection, while Burger et al. reported both AAV2/1 and 2/5 enhance the retrograde transport of expressed proteins [115, 123].

Despite phenotypic variations in terms of PD-related behaviors following AAV-mediated a-syn expression, the majority of AAV a-syn studies report greater than 80% AAV-mediated a-syn expression in SNc dopaminergic neurons. Further characterization of AAV capsid serotype transduction efficiencies in mature rodent brains will benefit efforts to consistently reproduce a-syn pathology. Also, the approval of multiple AAV serotype capsids for use in humans (AAV 1, 2, and 9) may provide insight on whether or not lessons learned from AAV-mediated transgene expression in rodents are applicable to humans [111, 124].

### AAV purification and titer

Potential sources for variability between AAV studies come from the AAV purification method used as well as the method to determine functional viral titers, which have been previously described [80]. Importantly, the method used to purify AAVs for *in vivo* injection could alter the functional titer, which is determined by the proportion of empty AAV capsids or AAV genome copies that are not encapsulated, both of which will be incapable of protein expression *in vivo* [125]. Since most AAV titers are determined via DNA dot blot or by qPCR, which do not report functional titers, better and more efficient methods to determine the functional titers or AAVs will need to be developed. Undoubtedly, advancements in AAV quality control and improvements in vector transduction efficiencies will lead to a newer generation of AAVs rodent models that consistently mimic key aspects of a-syn pathology in PD patients [27].

## 3. Appropriate controls, age, species, and sex differences in modeling AAV-mediated a-syn pathology in rodents

Following unilateral AAV injection into the rodent brain, there is significant spread to the contralateral hemisphere which makes it difficult to utilize the uninjected side as a control [126]. Furthermore, bilateral injections of AAV-a-syn to model PD in rodents are rarely employed[22, 69, 70]. Therefore, many studies utilize separate injections of control AAVs as empty vectors or encoding fluorescent reporters such as GFP or mCherry (Table 1). Since high titers AAV-GFP induce toxicity [16, 92, 112], proteins that form non-toxic oligomers, such as actin or collagen, might serve as a better control for AAV a-syn overexpression models [112].

Age is a critical factor when considering parameters when designing AAV-mediated a-syn expression experiments in rodents. Evidence for an influence of age on a-syn expression *in vivo* comes from a number of studies. Decreases in endogenous a-syn levels are present in 20-month old, but not in 10- and 2- month old male C57BL/6 mice [127]. Conversely, humans and non-human primates show increased a-syn levels with age [128]. Indeed, all human brain cell types exhibit changes in gene expression levels upon aging[129]. The effect of age on AAV a-syn-induced DA cell death has been compared between C57BL/6, and two outbred strains which are the rapid aging, SAMP8 and the age resistant, SAMR1 mice. Surprisingly, only C57BL/6 mice experienced cell death, indicating possible background susceptibilities or compensatory mechanisms, rather than age dependent a-syn pathology [66]. Similar differences related to strains are observed in rats. Specifically, Wistar rats are less susceptible to DA cell death when compared to Sprague Dawley (SD) rats [66].

A heavy species bias exists towards the use of SD rats as AAV a-syn models (Table 1). A specific preference for SD rats stems from the relative ease of performing behavioral paradigms in rats, the differential gene expression between species, and technical challenges related to injection of AAVs into the mouse SNc [130]. Surprisingly, there is also a sex bias towards the use of female SD rats, while male C57BL/6 mice are the chosen sex for AAV a-syn mouse models (Table 1). In humans, males are diagnosed with PD at a higher rate than women, and clinical symptoms have been shown to vary with sex [131, 132]. Given the central importance of sex differences in PD, future studies will need to focus on assessing differences in AAV-mediated a-syn expression, DA cell death, and PD-related behaviors between male and female rodents.

## 4. Additional tools to model a-syn pathology PD in rodents

Since the implication of a-syn in neurodegenerative diseases including Parkinson’s disease, Lewy body dementia, and multiple system atrophy, numerous molecular tools and animal models have been developed to study underlying mechanisms and efficacy of potential therapeutics. Although the vast majority of a-syn models utilize either transgenic mice, exogenous oligomers or PFFs, or viral vectors, recent data using combinations of these tools to induce PD has begun to show promise [71, 133]. In this case, each PD model varies in rate of a-syn spreading, degree of pathogenicity, and time course of PD progression.

Transgenic mice expressing either the mutant or wild-type strains of human a-syn were the first animal models to overexpress a-syn[134-136]. These models shows very little to no neurodegeneration in DA neurons of the SNc[137], but they do show neuronal inclusions similar to LB seen in human PD, as well as deficits in striatal dopamine, neuroinflammation, and prodromal behaviors including cognitive, sleep, and olfactory dysfunctions [1, 73, 138, 139]. Despite these caveats, a-syn overexpressing transgenic mice likely reflect pathological processes that occur before the onset of neurodegeneration, which makes this approach a useful tool to study the role of a-syn dysfunction during early-stage PD. Moreover, the expression of a-syn aggregates in transgenic mice may be a useful model for determining the efficacy of therapeutics in mediating or even reversing a-syn aggregation.

The use of oligomers or PFFs, either injected into the brain [71, 140, 141], or into the gut [76, 142] is a second tool that has recently gained traction and is increasing our understanding of the nature of a-syn spread within the brain. PFFs cause robust DA cell death and show extensive spread of a-syn pathology across brain regions [100, 143, 144]. Ser-129 phosphorylation of endogenous a-syn in response to extracellular a-syn fibrils exerts profound effects on the ability of a-syn to form toxic aggregates[143], and may be of particular relevance to the spread of a-syn pathology across brain regions. Injection of exogenous a-syn fibrils can elicit an immune response in microglia not present with monomeric a-syn [145]. Similar to AAV-mediated a-syn, PFF injections are regionally targeted and typically unilateral [146]. PFFs also mirror the widespread variability among results (DA neurodegeneration, changes in striatal dopamine, motor deficits) seen in AAV a-syn models [71, 80, 146]. Differences in size and structural homogeneity of PFFs cause variability in results. However, recent advancements in standardizing PFF generation and characterization will help overcome this barrier. Thus, PFF a-syn rodent models offer distinct insights into the seeding and propagation of toxic species in a-synucleinopathies, and a unique perspective of PD pathogenesis.

While we focus on AAV-mediated a-syn models in this review, the use of other viral vectors in rodents have also enabled an understanding of a-syn pathology. Lentiviruses (LVs) have genomes that are roughly twice as large as AAVs, allowing for a larger payload. However, disadvantages of LVs include lower transduction efficiency, higher toxicity, and viral incorporation into the host genome [147]. There are relatively fewer studies utilizing LVs that demonstrate large variabilities in DA neurodegeneration [148-151]. The formation of neuronal, cytosolic inclusions in LV a-syn models have been exploited to test efficacy of PD treatments such as parkin, glial-derived neurotropic factor (GDNF), or heat shock protein overexpression [152, 153]. Furthermore, *in vivo* LV overexpression of mutated a-syn has shown that oligomeric a-syn is a critical determinant of the degree of a-syn-induced pathology [151]. This inherent toxicity and the probability of spontaneous recombination have been mitigated with newer generations of LVs [154, 155]. Thus, LVs have indeed become useful tools in gene therapy developments as well as therapy efficacy [155]. Importantly, the payload of LVs allows for the study of larger PD-related genes such as *LRRK2*[147]. While current a-syn models favor the use of AAVs, the use of LVs to confirm therapeutic efficacy, promote gene therapy, and understand the mechanisms of PD relevant genes is critical for understanding PD from multiple perspectives.

In summary, no one tool is able to fully recapitulate the molecular and behavioral pathogenesis of a-syn seen in human PD, but selecting the appropriate tool will allow greater accuracy in modeling specific aspects of PD pathology. Furthermore, combinations of these aforementioned tools are beginning to show promise in recapitulating the complexities of human a-syn pathology [71, 133, 140, 156].

## 5. Using AAVs to understand the role of astrocyte-specific a-syn in PD

Astrocytes govern important aspects of brain function such as glutamate clearance, extracellular K^+^ buffering, regulation and maintenance of the blood brain barrier, the release of antioxidants and gliotransmitters, and synaptic physiology [157-159]. Interestingly, many of the genes implicated in the progression of PD, such as *ATP13A2*, *GBA1, LRRK2, PARK7, PINK1* and *PARKIN* are highly expressed in human astrocytes [22, 160-162], and human post-mortem brain studies, *in vitro*, as well as *in vivo* models of a-syn [90] suggest a significant role for astrocytes in a-syn pathology. Together, these reports suggest that astrocytes play a critical role in the pathogenesis of PD, and in the propagation of a-syn pathology during later stages of the disease.

There is some evidence that a-syn may have a physiological role in astrocytes that is distinct from its role in neurons. Astrocytes alter gene expression in response to a-syn aggregates [163], and primary astrocytes from *SNCA* KO mice display disrupted incorporation of fatty acids arachidonic acid and palmitic acid, which indicates a possible physiological role for the low amounts of endogenous a-syn in astrocytes [161]. Conversely, the overaccumulation of a-syn in astrocytes elicits both a loss of physiological functions and gain of toxic, inflammatory functions. Despite evidence for a-syn function in astrocytes, AAV a-syn models have not focused on astrocytes. In the next sections, we highlight key findings that are relevant to a-syn pathology in astrocytes and astrocyte dysfunction in AAV-a-syn based models. We also discuss potential caveats for studying astrocytes using AAV-mediated a-syn expression.

### Astrocytic uptake and transfer of a-syn

Astrocytic inclusions containing aggregated a-syn have been observed in post-mortem brain samples of PD patients [3, 161, 164] and in human derived astrocyte cultures [104, 162, 165-167]. One theory is that astrocytes take up a-syn following extracellular secretion by neurons, which results in the formation of abnormal inclusions of a-syn in astrocytes. In support of this idea, different mechanisms of astrocytic uptake of a-syn in rodents using *in vitro* and *in vivo* models have been proposed [90, 168-170]. Following uptake by astrocytes or neurons, a-syn transfer may occur via prion-like spreading [39, 168], tunneling nanotubes [166, 171, 172], exosomes [173], or by endocytosis of dead neurons [174, 175]. Interestingly, Loria *et al* established that in neuron and astrocyte co-cultures, the fibrillary form of a-syn can be transferred from neuron to astrocyte, astrocyte to astrocyte, but not from astrocyte to neuron [167]. In the same study, fluorescently labelled a-syn fibrils localized to astrocytic and neuronal lysosomal compartments for degradation *in vitro*; however, only astrocyte and microglial cells showed lysosomes containing a-syn in organotypic cultures, consistent with other a-syn models [167]. Thus, astrocytes appear to not only contain abnormal a-syn during PD, but may also possess specialized mechanisms for the internalization and degradation of a-syn, which makes these cells a potentially important, but understudied cell type in the context of PD.

### Potential astrocyte dysfunctions from a-syn pathology

Studies have also begun to elucidate potential mechanisms that underlie astrocyte dysfunction in a-synucleinopathies. One interesting finding in this regard is that the abnormal accumulation of a-syn in astrocytes results in altered glutamate clearance [176]. Utilizing a transgenic mouse model with mutant A53T and WT a-syn directly targeted to astrocytes, another study has shown that the astrocytic glutamate transporters, GLT-1 and GLAST were downregulated [177]. In support of these previous studies, oligomeric a-syn from erythrocyte-derived extracellular vesicles of PD patients has been shown to co-localize with GLT-1 in astrocytes and is associated with a reduction in glutamate clearance, possibly due to the inhibition of GLT1 function [170].These findings together suggest that a-syn accumulation in astrocytes can significantly alter glutamate neurotransmission in the brain, which has important implications for neurodegeneration.

Finally, the role of astrocytes in neuroinflammation has only recently become an area of intense focus. Astrocytes actively participate in the degradation and clearance of a-syn [178]. Oligomeric and nonfibrillized a-syn deposition in astrocytes lead to activation of microglia *in vitro*[179] and *in vivo* [102, 177]. Moreover, release of cytokines such as TNF-α and IL-β from astrocytes initiate apoptosis in dopaminergic neurons[180]. In advanced stages of neurodegeneration, astrocytes are reactive and engulf a-syn aggregates, synapses, and dead cells [91]. Analogously, in healthy murine astrocytes, overaccumulation of amyloid-beta triggers an immune response [181]. Yet, neuroinflammation in the AAV a-syn model has largely been investigated in the context of microglia.

In sum, a large body of evidence suggests that astrocytes play a critical role in the pathogenesis of PD, and particularly in the pathological effects of a-syn. Targeting AAV-a-syn to astrocytes can therefore serve as a valuable tool to model the specific effects of a-syn on astrocytic function *in vivo*. In this regard, several AAV serotypes are capable of astrocyte transduction, including AAV 2/1, 2/5, 2/8, and 2/9 [118, 119, 182-185]. Recently, AAV 2/6 was shown to preferentially transduce astrocytes (>90%) in the barrel cortex of male SD rats[186], but none of the studies employing AAV 2/6 in Table 1 report expression of a-syn in GFAP labeled astrocytes. The use of AAV compatible astrocyte-specific minimal promoters such as GFaABC1D[187, 188] or the use of Flex AAV constructs to express a-syn in large populations of astrocytes from astrocyte-specific Cre mice [189] can provide novel insights into the role of astrocytes in propagating a-syn pathology. In addition, using signal sequences that target a-syn to subcellular organelles such as astrocytic mitochondria[188] could allow us to study how a-syn affects subcellular compartments of astrocytes with unprecedented precision.

## 6. Conclusion

Based on two decades of experiments, we now know that the expression of a-syn with AAVs in rodents recapitulates several aspects of PD. AAV-mediated a-syn expression causes defects in striatal neurochemistry that precede SNc DA loss, induces the formation of phosphorylated α-syn aggregates throughout the nigrostriatal pathway, and results in neuroinflammation as well as the activation of glia. AAVs possess a plethora of customizable features that include promoters, enhancers, inclusion of fluorescent tags, and serotype of viral capsid in which the construct is packaged. While the expression of a-syn using AAVs in rodents has undeniably expanded our understanding of the molecular pathology in PD and other a-synucleinopathies, the very same customizable features that make AAVs an attractive model, have also led to inconsistencies between various studies. Undoubtedly, future advancements in AAV quality control and improvements in vector transduction efficiencies will lead to a newer generation of AAVs rodent models that consistently mimic the key aspects of a-syn pathology in PD patients. Based on the emerging role of astrocytes during PD pathogenesis, an exciting new direction in PD research would be to apply AAVs as a tool to specifically understand the role of astrocytes in propagating a-syn pathology. We envision that lessons learned from two-decades of experiments utilizing AAV-a-syn to model PD in rodents will enable us to apply this knowledge towards understanding the critical role of astrocytes in propagating a-syn pathology during PD.
